# Automatic Recognition and Analysis of Balance Activity in Community-Dwelling Older Adults: Algorithm Validation

**DOI:** 10.2196/30135

**Published:** 2021-12-20

**Authors:** Yu-Cheng Hsu, Hailiang Wang, Yang Zhao, Frank Chen, Kwok-Leung Tsui

**Affiliations:** 1 School of Data Science City University of Hong Kong Kowloon Hong Kong; 2 School of Design The Hong Kong Polytechnic University Hung Hom Hong Kong; 3 School of Public Health (Shenzhen) Sun Yat-sen University Guangzhou China; 4 Department of Management Sciences City University of Hong Kong Kowloon Hong Kong; 5 Grado Department of Industrial and Systems Engineering Virginia Polytechnic Institute and State University Blacksburg, VA United States

**Keywords:** fall risk, balance, activity recognition, automatic framework, community-dwelling elderly

## Abstract

**Background:**

Clinical mobility and balance assessments identify older adults who have a high risk of falls in clinics. In the past two decades, sensors have been a popular supplement to mobility and balance assessment to provide quantitative information and a cost-effective solution in the community environment. Nonetheless, the current sensor-based balance assessment relies on manual observation or motion-specific features to identify motions of research interest.

**Objective:**

The objective of this study was to develop an automatic motion data analytics framework using signal data collected from an inertial sensor for balance activity analysis in community-dwelling older adults.

**Methods:**

In total, 59 community-dwelling older adults (19 males and 40 females; mean age = 81.86 years, SD 6.95 years) were recruited in this study. Data were collected using a body-worn inertial measurement unit (including an accelerometer and a gyroscope) at the L4 vertebra of each individual. After data preprocessing and motion detection via a convolutional long short-term memory (LSTM) neural network, a one-class support vector machine (SVM), linear discriminant analysis (LDA), and k-nearest neighborhood (k-NN) were adopted to classify high-risk individuals.

**Results:**

The framework developed in this study yielded mean accuracies of 87%, 86%, and 89% in detecting sit-to-stand, turning 360°, and stand-to-sit motions, respectively. The balance assessment classification showed accuracies of 90%, 92%, and 86% in classifying abnormal sit-to-stand, turning 360°, and stand-to-sit motions, respectively, using Tinetti Performance Oriented Mobility Assessment-Balance (POMA-B) criteria by the one-class SVM and k-NN.

**Conclusions:**

The sensor-based approach presented in this study provided a time-effective manner with less human efforts to identify and preprocess the inertial signal and thus enabled an efficient balance assessment tool for medical professionals. In the long run, the approach may offer a ﬂexible solution to relieve the community’s burden of continuous health monitoring.

## Introduction

Falls prevail among the aging population, and led to more than $30 billion in direct medical costs in 2015 [1]. Around 55% of unintentional injury deaths among older adults in the United States are due to falls [2]. Falls pose a threat to the physical and psychological aspects of older adults’ health [3,4]. It is critical to identify older adults at the risk of falls and take interventions in advance [5].

Fall risk factors can be grouped into extrinsic (environmental) factors and intrinsic factors (age, health status, and other factors derived from the human). Outpatients and community dwellers usually suffer less from illnesses; consequently, intrinsic mobility and balance are the most discriminative intrinsic indicators of falls [6]. Mobility and balance assessment tools, such as the Balance Evaluation Systems Test (BESTest) [7], the Tinetti Performance Oriented Mobility Assessment (POMA) [8], and the Berg Balance Scale (BBS) [9], have been popular in community and outpatient settings to assess the mobility and balance aspects of individuals [6]. Along with the use of these instruments, a trained health care professional observes the participants’ motion(s) as they complete a series of tasks (eg, sit-to-stand, turning 360°, and stand-to-sit) and scores their performance based on medical expertise.

Nevertheless, such assessment tools carry disadvantages that prevent older adults from undergoing frequent fall assessments [10]. First, many mobility and balance assessments, such as the BESTest [7], the Tinetti POMA [8], and the BBS [9], take from 15 to 35 min to complete [6,11], which is time consuming and burdensome to implement on a community-wide scale. Second, traditional assessments heavily rely on observations made by medical professionals [6,10]. Such resource-demanding assessments become unaffordable, which is a phenomenon commonly observed in Hong Kong [12,13]. A review [14] reported that over one-third of elderly services units fail to fill their physiotherapist and occupational therapist vacancies. In short, conducting mobility and balance assessment requires time, human resources, and financial availability, which further discourages older adults from frequently checking their fall risk.

Thanks to the rapid development of information technology, the sensor provides a practical solution to this predicament nowadays. Commercial sensors (eg, accelerometer and gyroscope) are affordable to most community service sites or health care agents [15,16]. These sensors provide an objective measurement of motion that can support health professionals’ decision making or act as a preliminary screening tool in the absence of professionals. Current research [17-19] has proven the utility of this approach by validating sensor-based assessments with clinical mobility and balance assessment tools. Researchers [20-25] have quantified and analyzed several sensor features that indicate motion and balance capability insufficiency. Halilaj et al [26] emphasized the importance of interpretability of sensor features and the model in this application for health care professionals and individuals. The majority have adopted statistical models to provide a statistical explanation of the work [10,16,27]. Some recent research [28-30] has argued that though the neural network approach might not be as interpretable as the previous research, it provides an accurate prediction. Sensor technology offers a quantitative method of studying human mobility and balance and coincides with clinical mobility and balance assessments.

Although there are advantages to the use of sensor technology, it is also important to acknowledge the residing problems. Ideally, individuals can perform these sensor-based assessments independently. In practice, irrelevant signals that come before and after performing the testing motion might be recorded. Therefore, an additional process to remove those irrelevant signals becomes necessary. Existing studies have relied on an additional research assistant’s manual observations [28,31-33] or crafted features specific to motion [34] for identifying the motion signal, which will be assessed in the sensor-based assessments. In addition, although crafting features and building a heuristic algorithm [34,35] to detect motion provide a scientific solution, it is limited to specific, well-studied activity. This limitation makes detection hard to be generalized and transferred to different motion analyses. The additional manual efforts for identifying sensors or recording signals discourage health care professionals and community users from adopting sensor-based approaches [36].

Human activity recognition (HAR) is a practical remedy to relieve this burden. The majority of developed methods adopt machine learning models [37-39] or deep learning [40-45] to build on and validate renowned public data sets, such as the University of California, Irvine (UCI)-HAR [46] and UniMib SHAR [47], that target healthy adults aged 30 years or less. In contrast, only a few research works [48,49] focus on older adults. These studies [37-39] and reviews [43] claim that HAR techniques can be applied for health care purposes. Nonetheless, to the best of our knowledge, there is no existing work that explicitly delineates the combination of HAR and sensor-based mobility and balance assessments as completely automated assessments.

**Figure 1:**
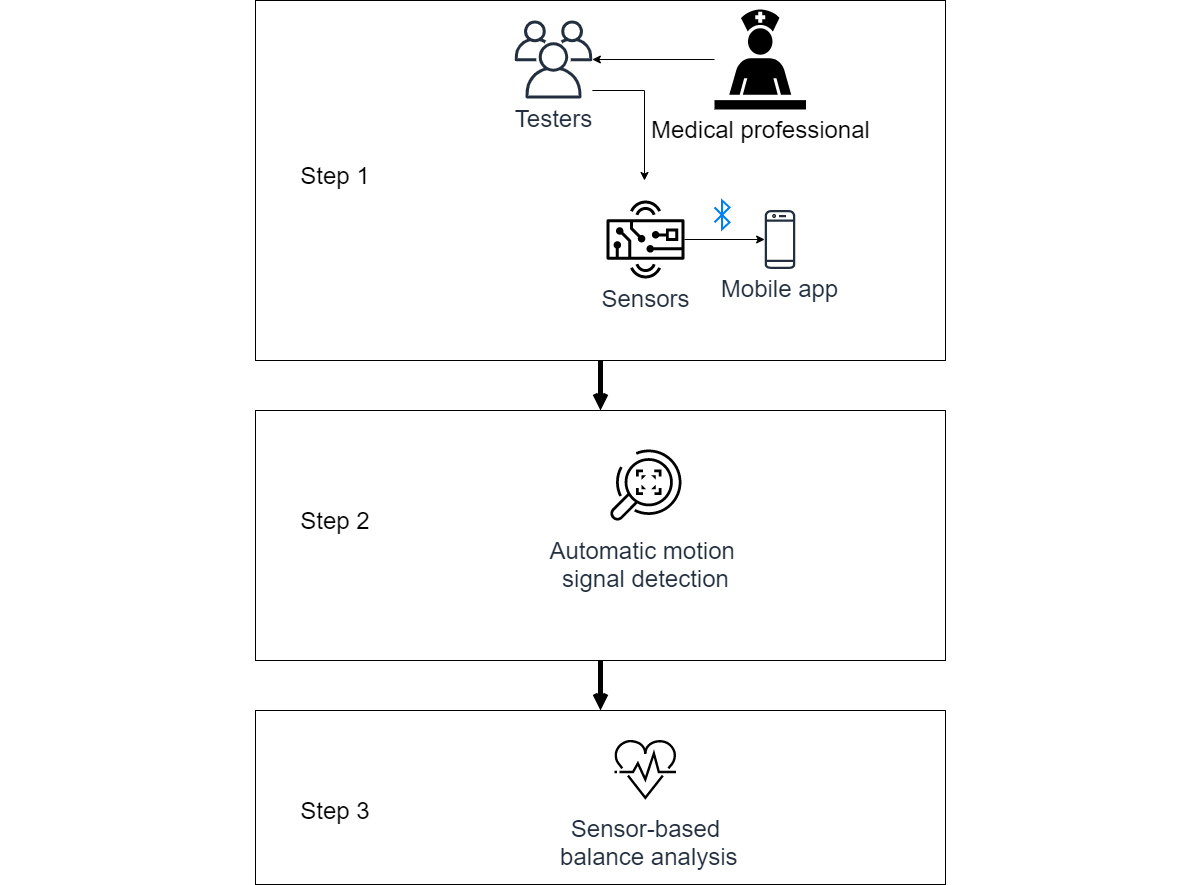
Overview of the developed framework.

This study illustrates an automatic sensor-based framework (Figure 1) of motion and balance assessment. We hypothesized that the motion detection method is a solution for better automating sensor-based balance assessments. This framework resolves the requirement of human input in the preprocessing stage and completes the whole automation data pipeline. The developed framework aims to solve a simplified motion detection problem by leveraging the application scenario and a motion evaluation according to the Tinetti POMA-B grading standard. This study embodies this framework with deep-learning motion detection and sensor-based mobility and balance assessment. The major contributions of the proposed method are two-fold. First, few existing studies integrate motion detection and sensor-based balance assessment to form the data analysis pipeline, to the best of our knowledge. Second, the proposed method requires less human effort to identify and preprocess the inertial signal and enable a more efficient sensor-based balance assessment tool for medical professionals. This framework offers a flexible automatic solution to relieve the community’s burden during large-scale implementation, such as long-term balance monitoring.

## Methods

### Recruitment

In total, 59 community-dwelling elders (19 males and 40 females; mean age = 81.86 years, SD 6.95 years) participated in our study from September 2019 to December 2019. Participants with behavioral problems (eg, violence), unstable mental status (eg, paranoia), communication problems (eg, dialect), or severe hearing impairment were excluded from the study. Written informed consent was obtained from all participants before performing data collection. The imbalanced gender distribution was a direct reflection of the outnumbered male participants enrolling in community services [50]. Among the participants, six had experienced falls within the past year. A detailed breakdown of the demographics is shown in Table 1. This study was approved by the Research Ethics Committee of City University of Hong Kong (reference no. 3-2-201803-02).

**Table 1:** Demographics of all participants and group difference according to Tinetti grading items (N=59; 19 males and 40 females; mean age 81.86 years, SD 6.95 years).

Score		Age (years), mean (SD)	Males:females (n)
**Sit-to-stand**			
	<4	86.64 (6.05)	6:8
	4	80.37 (6.79)	13:32
**Turning 360°**			
	<2	85.55 (5.88)	6:5
	2	81.02 (6.90)	13:35
**Stand-to-sit**			
	<2	87.00 (5.15)	5:9
	2	80.26 (6.65)	14:31

The score followed the Tinetti POMA grading guideline [8]. The Tinetti POMA grading guideline is the mobility and balance assessment outcome used in this study. Age affected the Tinetti POMA performance, but gender did not. Age showed a significant difference between the sit-to-stand score=4 and <4 groups (Student *t* test, *P*<.001). Age also showed a significant difference between the stand-to-sit score=2 and <2 groups (Student *t* test, *P*<.001). There was no gender difference in all of these tasks (Fisher exact test *P*=.99, 0.44, and 0.50 for sit-to-stand, turning 360°, and stand-to-sit motions, respectively.)

### Experiment Protocol

In this step (Step 1 in Figure 1), balance assessments were conducted with an inertial sensor. Each participant was asked to perform three actions: standing up from an armless chair, turning around, and sitting down in the armless chair. There were 59 recorded inertial signals for each task from 59 participants. The three motions are commonly used in many functional assessments. All the three motions are adopted in the BBS [9], the Physical Performance Test (PPT) [50] and Tinetti POMA [8]; standing up and turning are adopted in the Fullerton Advanced Balance (FAB) [51] and the clinical Gait and Balance Scale (GABS) [52]; standing up and sitting down are adopted in the Postural Assessment Scale for Stroke Patients (PASS) [53], the Sensory Orientated Mobility Assessment Instrument (SOMAI) [54], and the Activity-based Balance Level Evaluation (ABLE) scale [55]. These three motions were considered sufficient to illustrate the idea of motion detection and evaluation in the developed framework.

**Figure 2:**
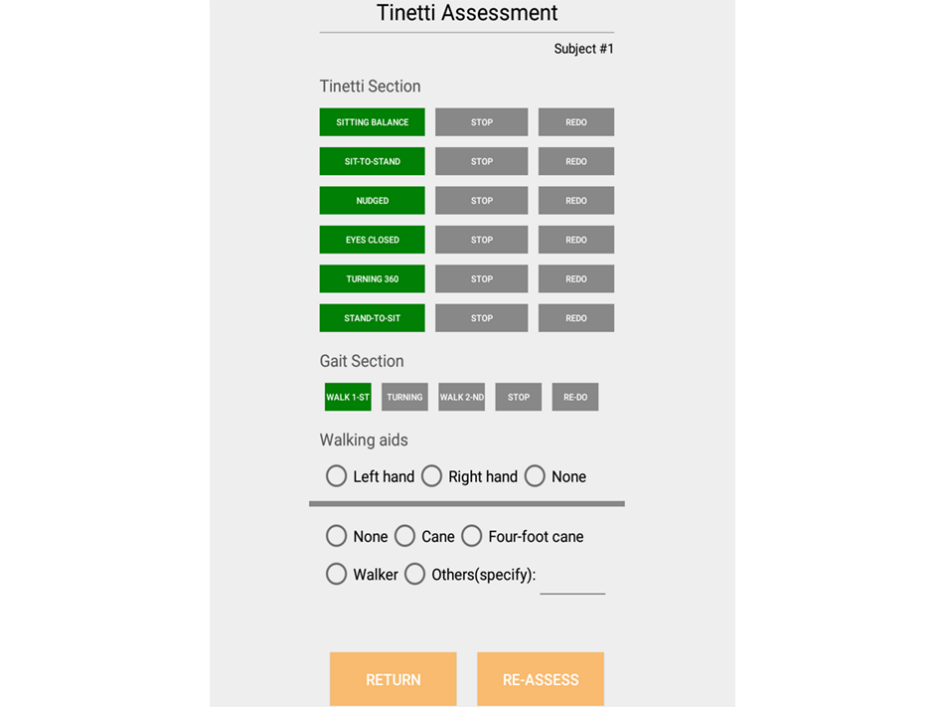
Screenshot of the data collection app.

A commercial inertial measurement unit (Wit-motion JY901B; including an accelerometer and a gyroscope at 3 axes with 16-bit resolution, sampling frequency 40 Hz, and a built-in Kalman filter) was attached on an elastic belt and placed on the L4 vertebra of each participant prior to each task. The inertial signal was transmitted via Bluetooth to the dedicated mobile application. The participants would then listen to the instruction and perform it accordingly. As soon as a participant initiated the task, a research assistant would press the button (green buttons and STOP buttons in Figure 2) on the app to mark the starting time and ending time, as shown in the clock on the mobile device. Figure 2 displays a screenshot of the data collection app.

Before starting the assessment, each participant was required to perform a trial. Sit-to-stand and stand-to-sit motions were conducted using a straight-backed armless chair. Participants were asked to complete the turning task in a fast yet safe manner according to their judgment. These motions were conducted in line with Tinetti POMA guidelines. Participants were asked whether they needed a break for rest, but none needed it.

### Automatic Motion Detection

In this part (Step 2 in Figure 1), we aimed to determine the starting and ending times for a known task from inertial signals containing only a known task (sit-to-stand, turning 360°, and stand-to-sit) and nontask activities. Since some participants needed extra time to comprehend and respond to the instruction, some recordings included pretask records. In addition, it also took a few seconds for users to stop the recording after the participants completed the motion. Therefore, some sensor signals irrelevant to the motion might be included in the data. Since the motion to be analyzed was known in the prescribed mobility and balance assessment list, we only needed to detect the motion. The developed framework leverages these facts to reduce the complexity of motion detection to a simpler binary classification problem. We built separate classifiers for each task motion, allowing each piece of signal that contained a known task (ie, sit-to-stand, turning 360°, or stand-to-sit) to go through the data pipeline separately, as introduced below.

#### Preprocessing

The acceleration signal was detrended by subtracting the mean of the acceleration at each axis. The gyroscope signal was converted into the angular displacement from the starting point by integration. The gyroscope’s drift issue was neglected as each task took less than 30 s to complete, and the drift rate is at 0.05°/s according to the product specification. Subsequently, each task signal was segmented from the preprocessed task signals into 0.75 s sliding windows with a step size of 0.03 s. The different sliding window sizes are examined and discussed in the Results and Discussion sections. The label of the sliding window segment is defined as the percentage of the class it covers. For example, a sliding window segment that contains 0.5 s of a sit-to-stand task and 0.25 s of the nontask motion would be labeled as 0.67 for the class sit-to-stand and 0.33 for the class nontask motion.

#### Convolutional LSTM Motion Detector

**Figure 3:**
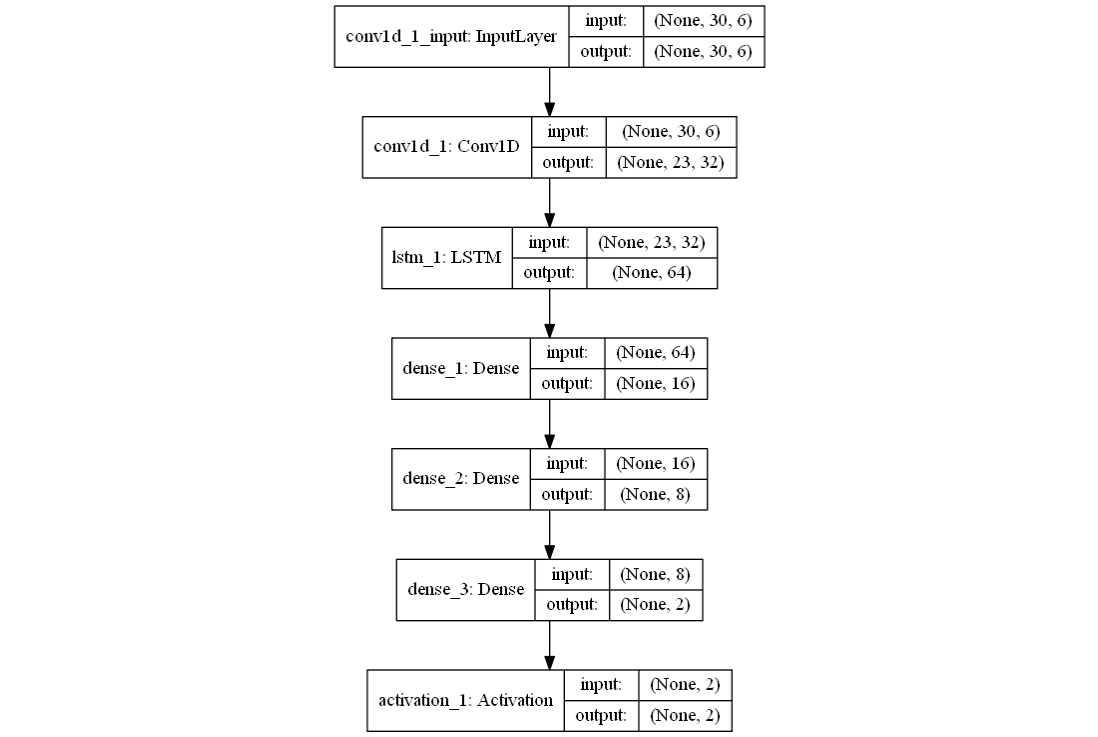
Structure of a convolutional LSTM used in motion detection. LSTM, long short-term memory.

A convolutional long short-term memory (LSTM) neural network was built for detecting the starting and ending times for each task. The motion detection model takes the raw acceleration and angular displacement at three axes in the 0.75 sliding windows as input. The convolutional layers and LSTM layers can extract the temporal human dynamic inertial signals to construct the classification. The overall network structure is shown in Figure 3. This structure was inspired by a previous study [56], which used convolution networks to obtain the feature maps and LSTM structure to learn the temporal pattern.

The convolution layer took the preprocessed sliding window segment as input and performed a convolution operation with 32 filters, kernel size 3. The LSTM layer is a specific implementation of the recurrent neural network that takes the previous state information into the current calculation. Finally, the densely connected layer operates by applying the activation function of the dot operation of weights. The first two dense layers did not apply any activation function, and the SoftMax function activated the last dense layer to calculate the percentage of labels containing and not containing the task.

#### Postprocessing and Labeling

**Figure 4:**
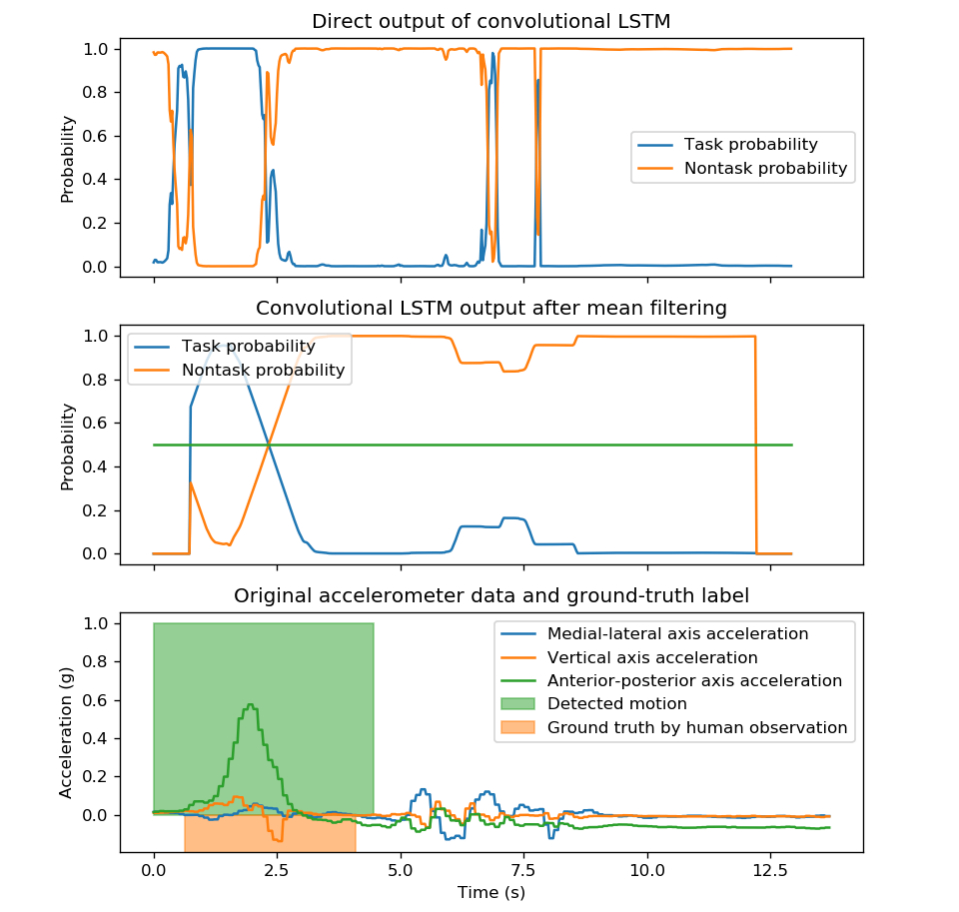
Original signal and output of the convolution LSTM network before and after processing. LSTM: long short-term memory.

The direct prediction of the convolutional LSTM neural network appeared to be noisy, as shown in the top image of Figure 4. This phenomenon was also observed in a similar network structure in HAR [56]. Therefore, the following postprocessing was performed to determine the beginning and end of the task.

A mean filter was used to filter the convolutional LSTM neural network output with window sizes of 1, 1.25, and 1.5 s. These mean filter sizes used in this section are examined and discussed in the Results and Discussion sections. The output is shown in the middle image of Figure 4. Subsequently, the candidate intervals were identified according to the following two rules:

1. Beginning of the interval: The closest peak of nontask probability before the point where task probability starts to be greater than 0.5
2. End of the interval: The first peak of nontask probability after the point where task probability starts to be less than 0.5.

Peak detection was conducted using the SciPy package [57] with 0.5 thresholds.

Typically, only an interval would be identified. If more than one interval was identified, the following rules were applied accordingly to the different tasks. For sit-to-stand and stand-to-sit tasks, the time interval that contained the greatest anterior-posterior (AP) acceleration range was assigned to when the participant performed the motion. For the turning 360° task, intervals with cumulative angular movement from a starting time less than 360° along the turning axis (vertical axis) were selected. The final result is illustrated in the bottom image of Figure 4.

These rules were inspired from the biomechanical point of view for human motion [58], where AP acceleration drastically changes during the sit-to-stand or stand-to-sit motion. The assumption is that only one movement prevails in the signal recordings, as stated previously.

### Sensor-Based Mobility and Balance Assessment

After the signal was automatically annotated, sensor-based mobility and balance assessment was performed to evaluate the corresponding motion (Step 3 in Figure 1). Sensor features that have been used in several previous studies were extracted from the detected signal and evaluated in relation to its Tinetti POMA-B grading items. This part consisted of feature extraction and prediction modeling to achieve the goal.

#### Feature Engineering

#### Sit-to-Stand and Stand-to-Sit Tasks

The count of the acceleration peaks along the AP axis was extracted from the labeled data. Peak detection was conducted using the Scipy package [59] with the threshold 45% of the maximum value. A normal sit-to-stand transition usually shows one peak along the AP axis [58]. Multiple peaks imply the possibility that the motion was not smooth or multiple attempts were performed to achieve the task.

#### Turning 360° Task

The average turning speed along the vertical axis was extracted from the labeled data. Research [60] shows a significant difference in turning speed between fallers and nonfallers.

#### Prediction Modeling

Participants who did not receive full marks in the corresponding Tinetti POMA-B grading items were labeled as deviating from the norm in performing such tasks in this study. The goal of mobility and balance assessment is to identify older adults with insufficient mobility and balance capability and intervene as early as possible. Therefore, we aimed to identify older adults whose balance evaluation score was different from that of healthy adults (ie, full marks in the balance assessment). The sensor features introduced above were used to predict the corresponding Tinetti POMA-B grading items. A detailed description is presented in Table 2, and the corresponding sensor feature distribution between two populations is tabulated in Table 3.

**Table 2:** Tinetti POMA-B^a^ task, grading items, and deviation from the healthy people criteria.

Task and grading item		Score	Deviation from healthy adults’ criteria	Feature
**Sit-to-stand**			Tinetti POMA-B total sit-to-stand score <4	AP^b^ acceleration peak count
	Arises from the chair	~0-2		
	Attempts to arise	~0-2		
**Turning 360°**			Tinetti POMA-B total turning 360° score <2	Average turning speed
	Turns 360° continuously	~0-1		
	Turns 360° steadily	~0-1		
**Stand-to-sit**			Tinetti POMA-B total stand-to-sit score <2	AP acceleration peak count
	Sits down	~0-2		

^a^POMA-B: Performance Oriented Mobility Assessment-Balance.

^b^AP: anterior-posterior.

**Table 3:** Sensor feature distribution between normal and deviation from healthy participants.

Task	Sit-to-stand AP^a^ acceleration peak count, mean (SD)	Turning 360° average turning speed (°/s), mean (SD)	Stand-to-sit AP acceleration peak count, mean (SD)
Healthy people	1.00 (0.29)	58.16 (19.22)	0.98 (0.15)
Deviating from healthy people	2.46 (2.02)	23.46 (13.96)	1.54 (0.75)

^a^AP: anterior-posterior.

A one-class support vector machine (SVM) [59], linear discriminant analysis (LDA), and k-nearest neighborhood (k-NN) from previous research works [31,61,62] were adopted in balance assessments.

### Evaluation Metrics

Accuracy and the area under the curve (AUC) were used to evaluate the performance of this work. Accuracy was defined as the percentage of observations classified into the correct class, as (TP + TN)/(TP + TN + FP + FN), where TP is the true-positive class being classified as positive, TN is the true-negative class being classified as negative, FP is the false-negative class being classified as positive, and FN is the false-positive class being classified as negative. The AUC was obtained from the area under the receiver operating characteristic (ROC) curve. The ROC curve plots the sensitivity [TP/(TP + FN)]) against the false-positive rate [1 – (TN/{TN + FP})] at different levels of thresholds.

The agreement between the mobility and balance assessment results using the human-annotated label and the motion detection method was evaluated using the McNemar agreement test. The null hypothesis states that the two results show agreement in classifying the mobility and balance assessment evaluation results. The test statistic was calculated by z^2^ = (n_12_ – n_21_)^2^/(n_12_ + n_21_), where n_12_ is the prediction result of when the manual label sensor feature shows positive but the motion detection label sensor feature shows negative. In contrast, n_21_ is the prediction result where the manual label sensor feature shows negative but the motion detection label sensor feature shows positive. The test statistic followed a chi-square distribution with 1 degree of freedom.

### Training and Testing Environment

Leave-one-subject-out (LOSO) cross-validation (CV) was used for training and testing. Each time, all the participants’ inertial measurements except the i-th participant were used for training the motion detection and sensor-based balance assessment, and the inertial measurements from the i-th individual were used for testing. The convolutional LSTM neural network was built and trained using Keras [63] with TensorFlow [64].

The computer used for training was an Intel E5-2670 CPU with Nvidia Tesla K20 and CUDA version 10.2 on a Linux system. The batch size was set at 200, and the stopping rule was set for no improvement after 20 epochs. The rest of the computation was conducted using SciPy [57] and NumPy [65].

## Results

### Motion Detection

Our motion detection methods yielded moderate accuracy in detecting sit-to-stand, turning 360°, and stand-to-sit tasks from 85% to 88% at different levels (see Table 4). The difference in classification accuracy did not significantly vary between different mean filter sliding window sizes. The developed motion detection method detected the same motion performed by the participant not observed in the training stage, because the LOSO CV withheld the participant’s motion signal as a testing set in each train and validation cycle. The training time and testing time showed that it took more than 1500 s to train the model, but detection was conducted within 0.3 s/participant.

**Table 4:** Accuracy of the automatic motion detection in different sliding windows.

Task	Accuracy (~Q1-Q3) mean filter 1 s sliding window (%)	Accuracy (~Q1-Q3) mean filter 1.25 s sliding window (%)	Accuracy (~Q1-Q3) mean filter 1.5 s sliding window (%)	Training time (s)	Testing time (s)
Sit-to-stand	87 (~82-95)	86 (~82-94)	85 (~78-93)	1681 (479)	0.21 (0.06)
Turning 360°	86 (~82-94)	86 (~79-95)	85 (~78-93)	2686 (495)	0.26 (0.09)
Stand-to-sit	88 (~85-94)	89 (~86-95)	88 (~83-94)	2186 (512)	0.24 (0.03)

### Sensor-Based Mobility and Balance Assessment

The sit-to-stand task motion was well detected and classified using the developed method. The detector had 85%-87% accuracy in detecting sit-to-stand motion that was not previously observed in the training set. The extracted feature, the peak count along the AP axis, exhibited a strong ability to discriminate between normal and abnormal motion in the sit-to-stand task through the k-NN method at 90% accuracy. The high accuracy could be attributed to the fact that multiple attempts to rise generate multiple peaks in the signals [58], which aligns with the Tinetti POMA grading criterion sit-to-stand transition. LDA showed the least discriminative capability because of the Gaussian distribution assumption. The result shows that the developed motion detection method and balance evaluation model can predict abnormal sit-to-stand motion.

The turning 360° task motion was detected using the developed method at an accuracy ranging from 85% to 86%. The classification showed 92% agreement with the professional opinion using k-NN in terms of the Tinetti POMA turning motion outcome. These results indicate that Tinetti grading items may correlate with the turning speed of the participants. Previous studies [66,67] have also reported that the turning speed is correlated with certain clinical mobility and balance assessment tools.

The stand-to-sit task motion was detected at 88%-89% accuracy by the developed method. Compared with the other two tasks, however, sensor-based prediction showed the least accuracy in predicting the functional assessment result, at 86% accuracy using the one-class SVM. This may be ascribed to Tinetti POMA’s grading to deduct a mark for participants who completed this task with the assistance of their arm, which occurred in the majority of the cases according to the report. This kind of information may not be revealed from the sensor signal, as the location of the sensor is at the participant’s lower back. Consequently, it could result in weaker performance in classifying the category of older adults.

## Discussion

### Principal Findings

The sensor features from the manual label signal and the motion detection method yielded little difference, which barely affects the sensor-based balance assessment results. Both sit-to-stand AP peak counts and stand-to-sit peak counts showed no statistical difference (Wilcoxon signed-rank test *P*=.42 and .45, respectively). The average turn speed showed discrepancy (*P*=.03) with a mean difference of 5.68°/s. In summary, the motion detection label features showed statistically no difference in the sit-to-stand and stand-to-sit motions but a slight difference in turning.

Using the manual label motion signal features or the detected motion signal features showed no statistical difference in classifying the Tinetti POMA outcomes in all circumstances (Table 5). The McNemar test was conducted to determine whether there is a difference in the classification outcome between the manual label and the presented motion detection. The results revealed that there is no statistical difference. The motion detection accuracy was satisfactory enough to ensure that the sensor features were not affected by the detection method. Consequently, practitioners could be comfortable with adopting the motion detection method instead of traditional laborious works. None of the models rejected the null hypothesis that both labels will yield the same result. This indicates that even though the motion detection method did not fully agree with the manual label, it still captures vital information in the sensor signal to predict mobility and balance assessments.

**Table 5:** Classification performance of sensor-based mobility and balance assessment using Tinetti-POMA-B^a^ criteria.

Task and metrics		One-class SVM^b^	LDA^c^	k-NN^d^
**Sit-to-stand**				
	AUC^e^ (%)	84	62	82
	Accuracy (%)	88	86	90
	*P* value of McNemar test	>.99	>.99	>.99
**Turning 360°**				
	AUC (%)	93	90	80
	Accuracy (%)	68	86	92
	*P* value of McNemar test	>.99	.25	>.99
**Stand-to-sit**				
	AUC (%)	60	72	56
	Accuracy (%)	86	83	80
	*P* value of McNemar test	.5	.5	.125

^a^POMA-B: Performance Oriented Mobility Assessment-Balance.

^b^SVM: support vector machine.

^c^LDA: linear discriminant analysis.

^d^k-NN: k-nearest neighborhood.

^e^AUC: area under the curve.

Previous research [36] has reported clinician concerns about the real-time application of sensor-based balance assessments. The testing results in motion detection indicated only a little delay in obtaining the results of the developed framework. Therefore, the presented implementation showed acceptance by the clinician in terms of time efficiency. In contrast, a survey [68] reported that most of the Hong Kong community-dwelling older adults perceive motion-analyzing systems as useful. Accordingly, we believe that the framework would receive acceptance from both clinicians and older adults.

### Limitations

There are four limitations of our study. First, we only analyzed sit-to-stand, turning 306°, and stand-to-sit motions rather than the complete mobility and balance assessment that covers comprehensive mobility and balance aspects. Nonetheless, motions used in this study have been frequently analyzed in several mobility and balance assessments to assess lower-body strength [69] and dynamic balance [70]. Other popular mobility and balance assessments, such as the Timed Up and Go (TUG) test [71], would be included in our future work. Second, instead of solving a HAR problem, this work leveraged the application scenario and broke it down into a detection problem, which only required an indication of the start and end times for the known task. This assumption helped the model to finesse the large variability in different motions in older adults. Third, we would like to expand the population and observations to enhance motion detection performance and balance assessments. In the framework discussed in this study, motion detection still relied on a certain amount of postprocessing, such as postprocessing after motion detection and feature engineering. This additional processing may become unnecessary with more observations for training. In addition, a greater sample size can also facilitate sensor-based balance evaluation with more complex modeling techniques and a one-off multiclass classifier to detect all the motions like in other works [28,30]. We can also observe that there are more healthy adults in the community than those with deviation from the healthy populations. A large sample size provides more in-depth insights into functional assessment with further complex feature engineering and feature selection methods. The last limitation is that the technology acceptance of this approach was not validated with the users. Though some related work with similar populations has shown good acceptance, as mentioned in the Discussion section, it is worth investigating how this approach can provide better user experience in the future plan.

### Conclusions

We presented human motion detection in a large-scale health care assessment. Existing works [72,73] on HAR have achieved satisfactory results in detecting activities from nonmovement validation on public data sets. Nonetheless, most of the public data sets are limited in the subject count and focus on healthy subjects than normal life. The network structure of this study was also inspired by several previous works [56,74] on HAR. This research aimed to provide a sensor-based balance assessment approach to clinical decision support with less human effort. We illustrated a framework using an inertial sensor for balance assessment. The major contributions are two-fold. First, few existing studies have integrated motion detection and sensor-based balance assessment to form the data analysis pipeline, to the best of our knowledge. Second, our method requires less human effort to identify and preprocess the inertial signal and enable a more efficient sensor-based balance assessment tool for medical professionals. This research examined the applicability of a deep-learning approach to detecting motion in the health care assessment context instead of general daily living.

This research illustrated the idea that functional assessment motions can be detected through HAR models. Therefore, the sensor data collection process can be conducted without additional labor if there is a sufficiently pretrained model in the future. Without the additional labor, the cost of sensor-based functional assessment can be reduced, providing more incentive to conduct large-scale implementation in identifying potential fallers in the community.

## Abbreviations

ABLE: Activity-based Balance Level Evaluation
AP: anterior-posterior
AUC: area under the curve
BBS: Berg Balance Scale
BESTest: Balance Evaluation Systems Test
CV: cross-validation
FAB: Fullerton Advance Balance
FN: false negative
FP: false positive
GABS: Gait and Balance Scale
HAR: human activity recognition
k-NN: k-nearest neighborhood
LDA: linear discriminant analysis
LOSO: leave-one-subject-out
LSTM: long short-term memory
PASS: Postural Assessment Scale for Stroke Patients
POMA-B: Performance Oriented Mobility Assessment-Balance
PPT: Physical Performance Test
ROC: receiver operating characteristic
SOMAI: Sensory Orientated Mobility Assessment Instrument
SVM: support vector machine
TN: true negative
TP: true positive
TUG: Timed Up and Go
